# Supplementary figures and images for: Role of Pcdh15 in the development of intrinsic polarity of inner ear hair cells
Source: PLoS Genet. 2025 Aug 13;21(8):e1011825. doi: 10.1371/journal.pgen.1011825 (PMC12370195; doi:10.1371/journal.pgen.1011825)

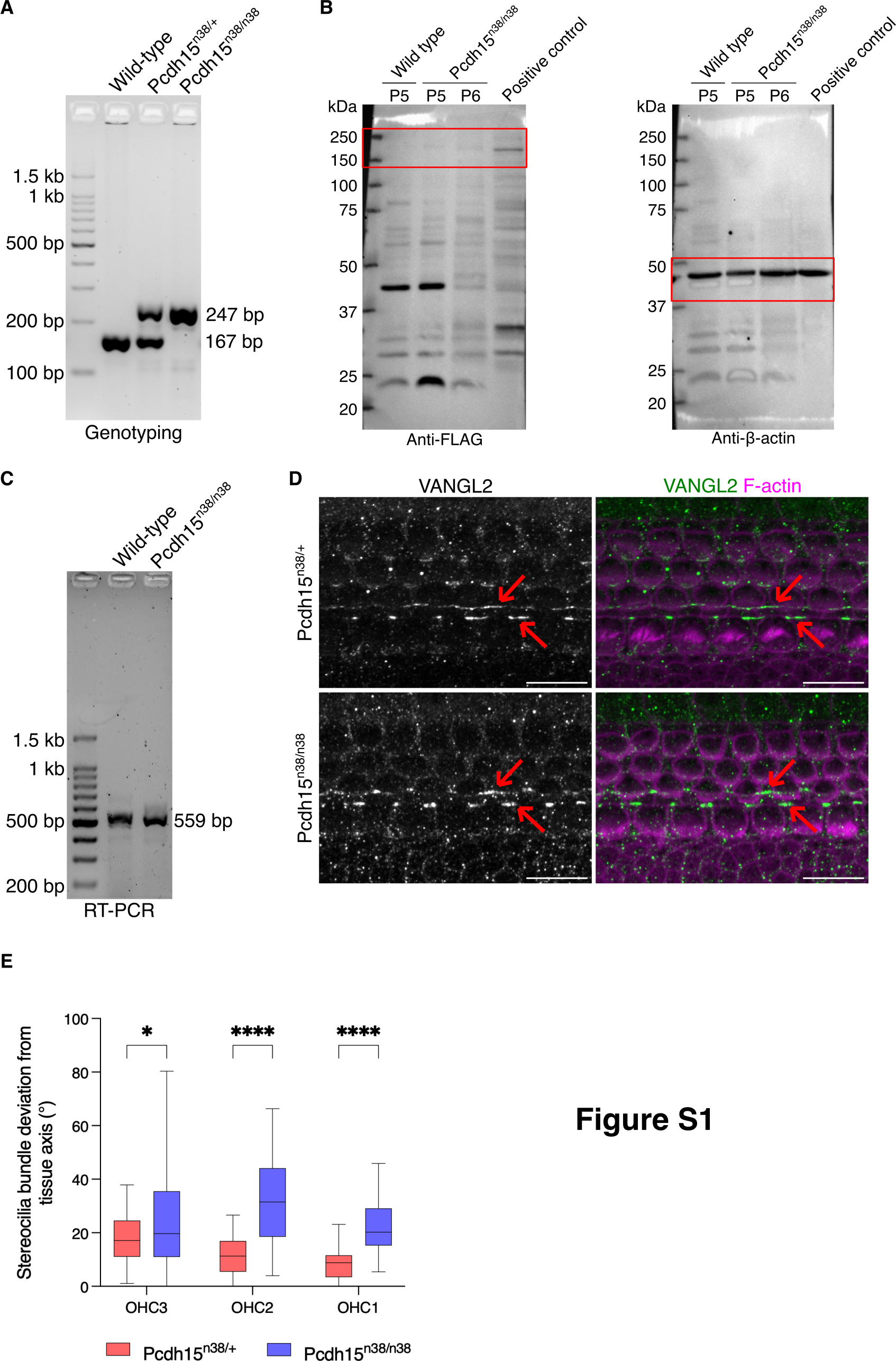

Supplement: S1 Fig — (A) A small fragment of Pcdh15 exon 38 was amplified in WT (167 bp) and Pcdh15n38/n38 (247 bp) animals for genotyping. (B) Full-length western blot of Fig 1D. (C) mRNA level for CD2 domain is normal in Pcdh15n38/n38 cochlea. (D) VANGL2 protein (green) localisation (highlighted with red arrows) is unperturbed in Pcdh15n38/n38 OC (E17.5, base). (Scale bar = 10 μm). (E) Stereocilia bundles are aligned normally with the tissue axis in Pcdh15n38/+ (pink), but significantly deviated in Pcdh15n38/n38 (slate blue) (P0, mid). (Two-way Anova with Tukey’s multiple comparisons test, ns = P > 0.05, * = P < 0.05, ** = P < 0.01, *** = P < 0.001, **** = P < 0.0001). (Sample size for OHC3/OHC2/OHC1, Pcdh15n38/+ = 69/67/72, Pcdh15n38/n38 = 69/68/69). (TIF) [file pgen.1011825.s001.tif]

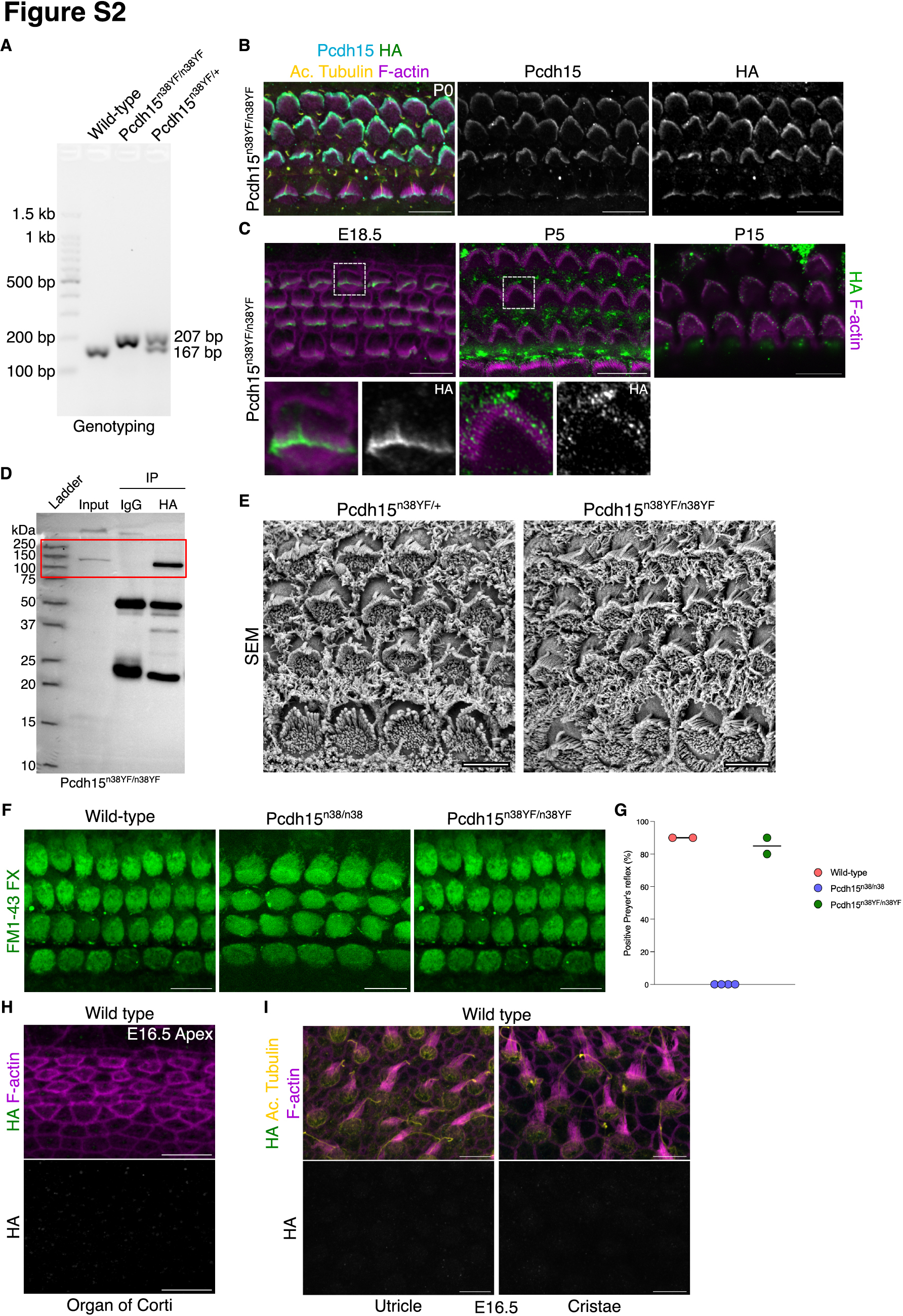

Supplement: S2 Fig — (A) Part of WT and Pcdh15n38YF/n38YF exon 38 are amplified in different sizes (167 bp and 207 bp, respectively) with the same primer pair for genotyping. (B) PCDH15-CD2-HA protein (green) is colocalised with PCDH15 (cyan) in the hair bundles (P0, mid). (Scale bar = 10 μm). (C) PCDH15-CD2-HA protein (green) is localised at the tip of stereocilia (magenta, marked with phalloidin) at E18.5 (mid). At P5 (mid), this expression is localised in all three rows of stereocilia and is refined more in P15 stereocilia (mid). (Scale bar = 10 μm). (D) Full-length western blot of Fig 2E. (E) SEM imaging shows normal hair bundle polarity in both Pcdh15n38/+ and Pcdh15n38YF/n38YF HC (P0, mid). (Scale bar = 5 μm). (F) MET channels are open in HC of Pcdh15n38/n38 and Pcdh15n38YF/n38YF mice at P5. FM1–43 FX dye (green) is used to detect open MET channels. (Scale bar = 10 μm). (G) Positive Preyer’s reflex response measurement shows no response in Pcdh15n38/n38 adult animals as compared to the comparable response in wild-type and Pcdh15n38YF/n38YF mice. (H-I) PCDH15-CD2-HA protein signal (green) is not present in wild-type mice organ of Corti (H) and utricle & cristae (I) at E16.5 stage (Staining controls). (Scale bar = 10 μm). (TIF) [file pgen.1011825.s002.tif]

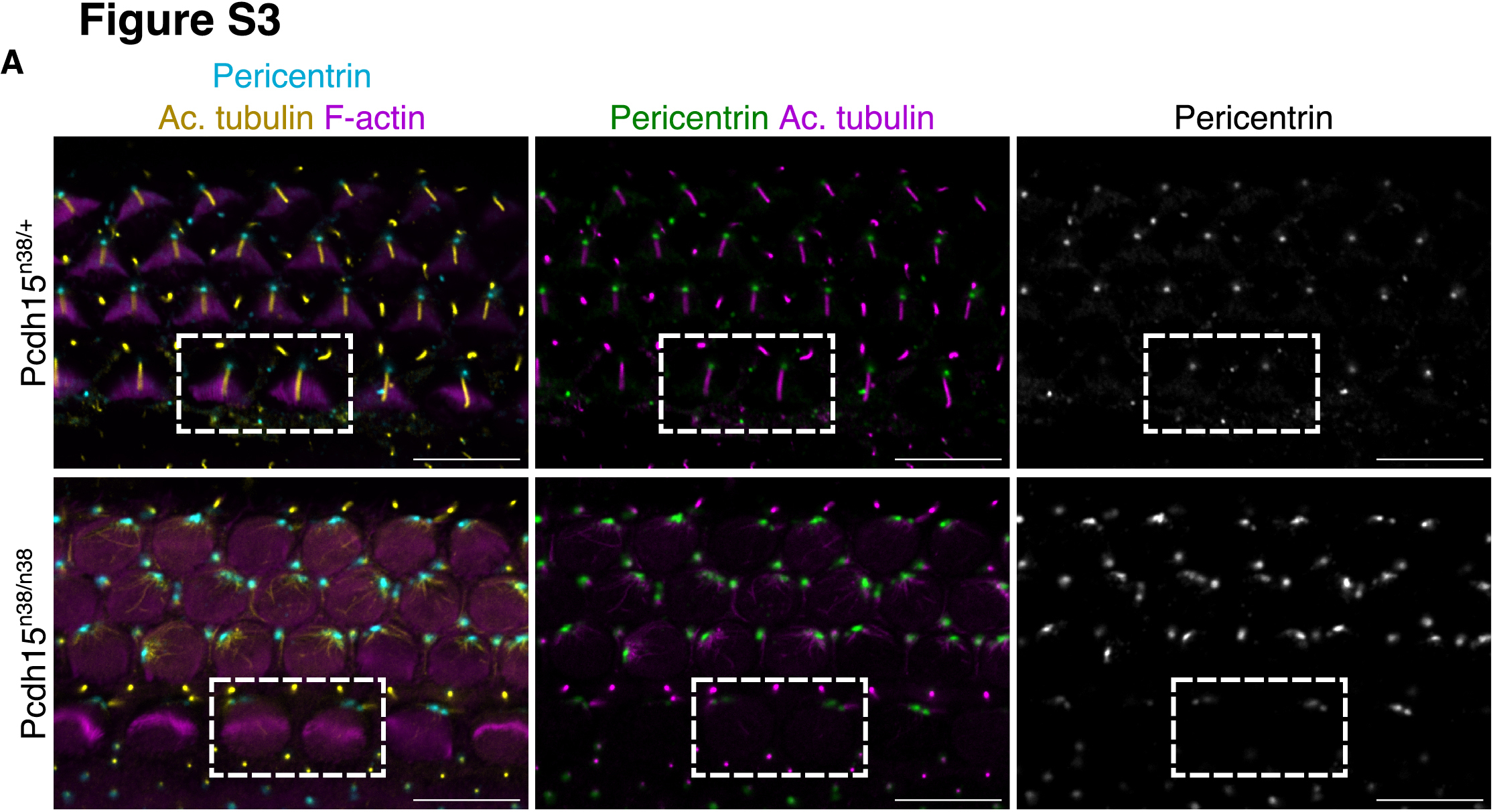

Supplement: S3 Fig — (A) A single optical section of the stained centrioles (marked with pericentrin) connected to the kinocilium (marked with acetylated tubulin) shows the absence of their orthogonal position to each other in HC of Pcdh15n38/n38 mice (E18.5, base). (TIF) [file pgen.1011825.s003.tif]

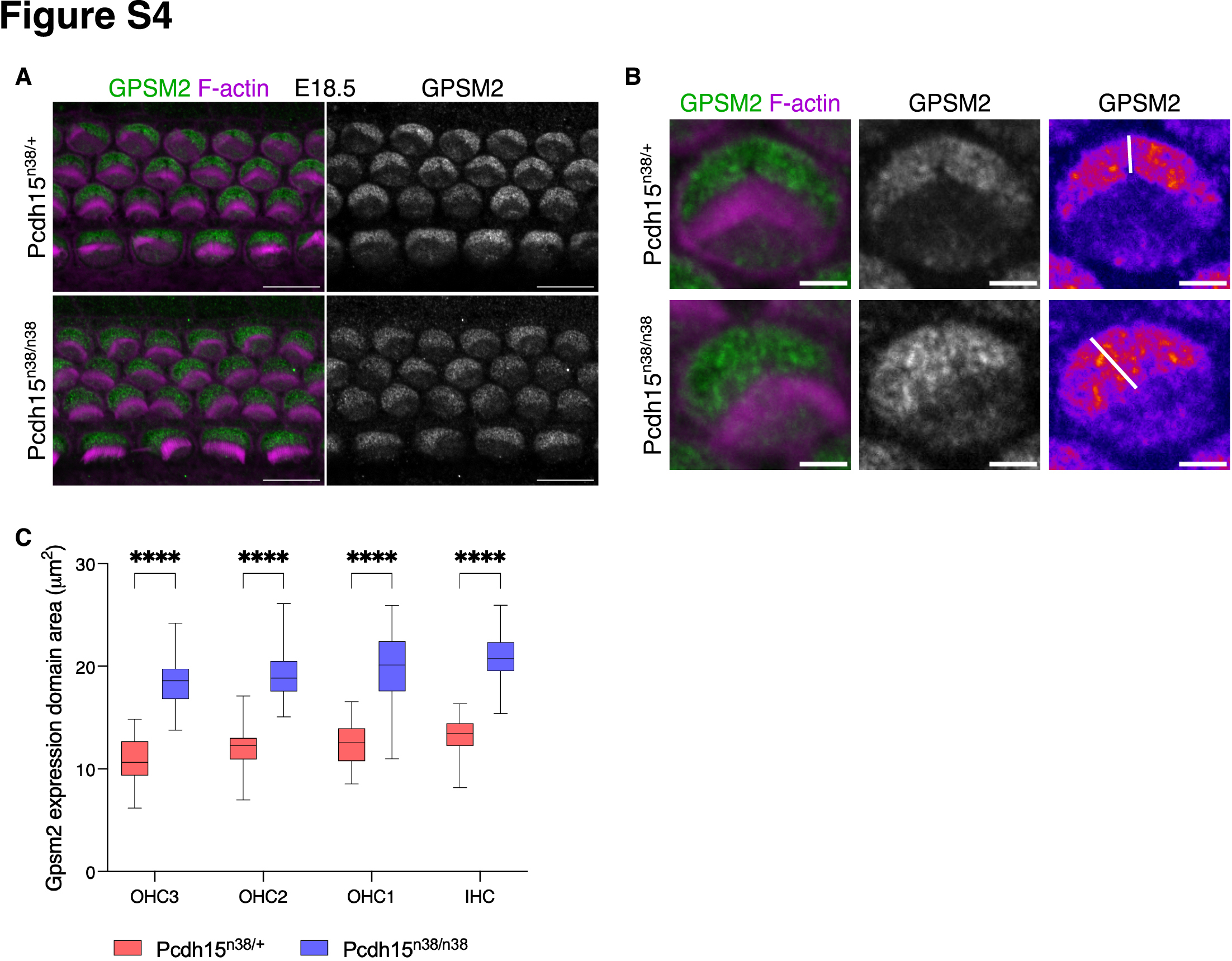

Supplement: S4 Fig — (A-B) GPSM2 expression domain (green) is extended medially in HC of Pcdh15n38/n38 mice (E18.5, base). (Scale bar = 10 μm & 2 μm). (C) In comparison to Pcdh15n38/+ mice, GPSM2 expression domain is spread significantly in HC of Pcdh15n38/n38 mice (E18.5, base). (Two-way Anova with Tukey’s multiple comparisons test, ns = P > 0.05, * = P < 0.05, ** = P < 0.01, *** = P < 0.001, **** = P < 0.0001). (Sample size for OHC3/OHC2/OHC1/IHC, Pcdh15n38/+ = 59/56/57/52, Pcdh15n38/n38 = 69/70/66/66). (TIF) [file pgen.1011825.s004.tif]

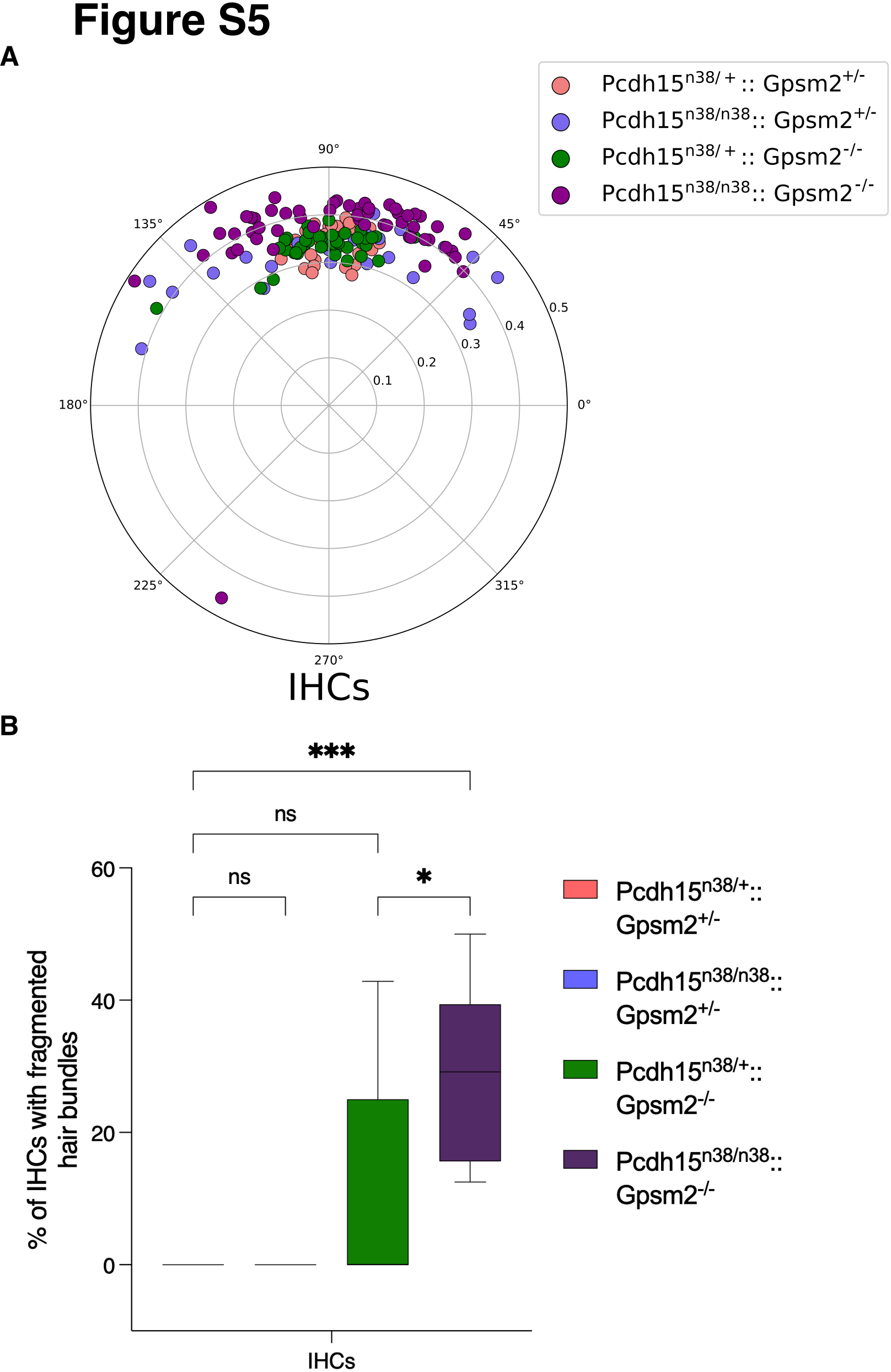

Supplement: S5 Fig — (A) In comparison to Pcdh15n38/+:: Gpsm2+/- (pink), the hair bundle polarity (polar projections) is perturbed in P1 IHC of Pcdh15n38/n38:: Gpsm2+/- (slate blue) and Pcdh15n38/n38:: Gpsm2-/- (dark magenta) OC (base). Hair bundle polarity is normal in Pcdh15n38/+:: Gpsm2-/- (green) OC. (B) Hair bundles are fragmented in IHC of only Pcdh15n38/n38:: Gpsm2-/- (dark magenta). (Ordinary one-way Anova with Tukey’s multiple comparisons test, ns = P > 0.05, * = P < 0.05, ** = P < 0.01, *** = P < 0.001, **** = P < 0.0001). (Sample size for IHC, Pcdh15n38/+:: Gpsm2+/- = 36, Pcdh15n38/+:: Gpsm2-/- = 42, Pcdh15n38/n38:: Gpsm2+/- = 24, Pcdh15n38/n38:: Gpsm2-/- = 54). (TIF) [file pgen.1011825.s005.tif]

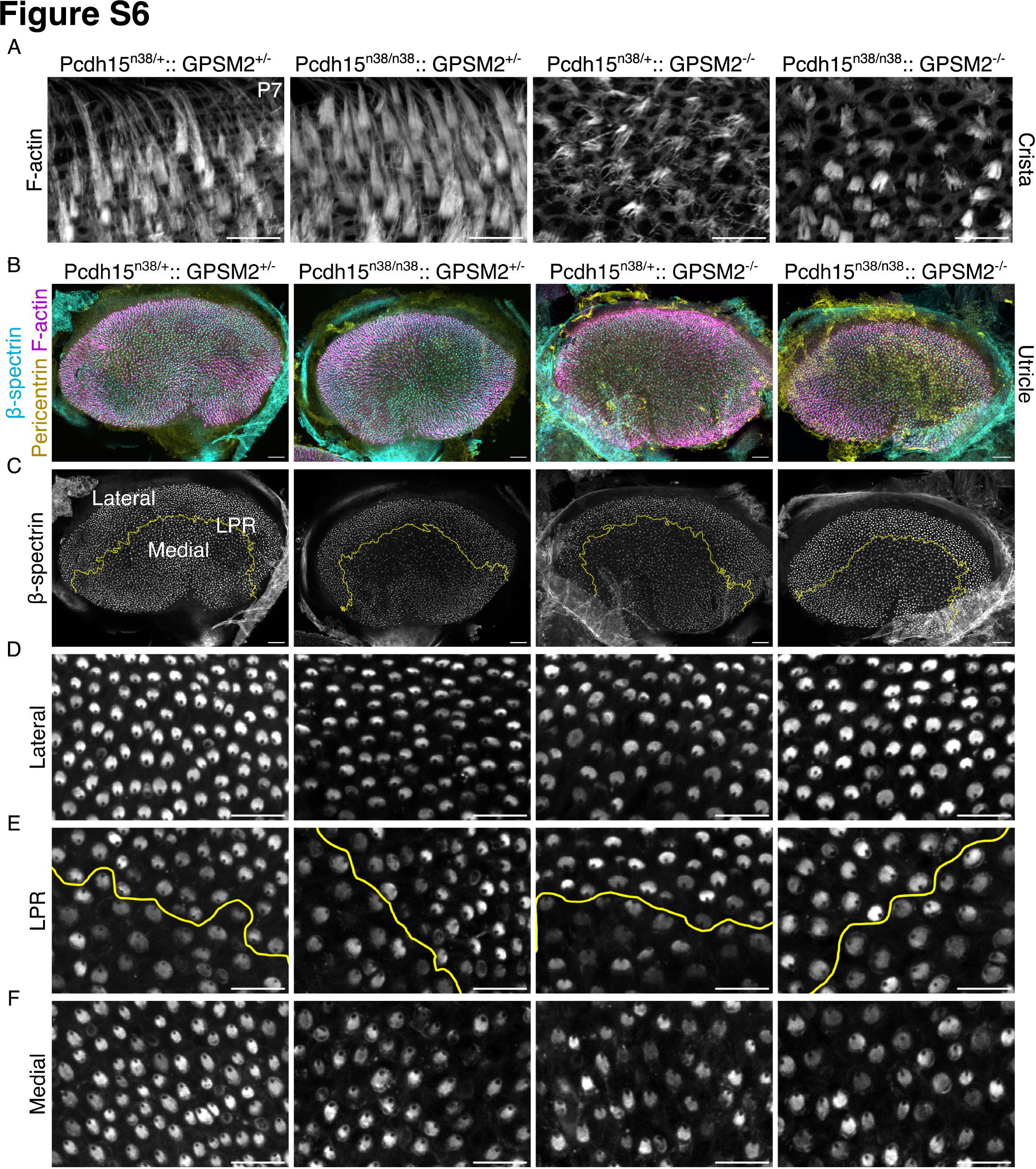

Supplement: S6 Fig — (A) F-actin (grey) shows smaller stereocilia bundles in the hair cells crista of Pcdh15n 38/+:: Gpsm2-/-, which is further reduced in Pcdh15n38/n38:: Gpsm2-/- mice, but not in Pcdh15n38/n38:: Gpsm2+/-. (P7, Scale bar = 20 μm). (B-F) Direction of hair cells in the (D) lateral, (E) LPR, and (F) medial region of the utricle shows comparable orientation in Pcdh15n38/n38:: Gpsm2+/-, Pcdh15n38/+:: Gpsm2-/- and Pcdh15n38/n38:: Gpsm2-/- mice as compared to littermate controls. (P7, Scale bar = 50 & 20 μm). (TIF) [file pgen.1011825.s006.tif]
