# Supplementary material for: Role of Pcdh15 in the development of intrinsic polarity of inner ear hair cells
Source: PLoS Genet. 2025 Aug 13;21(8):e1011825. doi: 10.1371/journal.pgen.1011825 (PMC12370195; doi:10.1371/journal.pgen.1011825)
Supplement: S1 Table — These include their specific primers, annealing temperature, and amplified product size. (DOCX) [file pgen.1011825.s008.docx]

**S1 Table**

| **Strain name** | **Primer name** | **Sequence (5'-3')** | **Annealing temperature** | **Product size** |
| --- | --- | --- | --- | --- |
| *Pcdh15^n38/+^* | 5 gt FW | GCCTGTTGGCTAGCTTCTGTCTATC | 55°C | WT = 167 bp, KI = 247 bp |
|  | 5 gt REV2 | CGTTAGCTCGGAGGTAGTTATGACG |  |  |
| *Pcdh15^n38YF/+^* | 5 gt FW | GCCTGTTGGCTAGCTTCTGTCTATC | 55°C | WT = 167 bp, KI = 207 bp |
|  | 5 gt REV2 | CGTTAGCTCGGAGGTAGTTATGACG |  |  |
| *Gpsm2* | FP1 | TTAGACTGTAATTGCTTATGTGC | 60°C | WT = 232 bp, KO = 350 bp |
|  | FP2 | TCCTCCATCTGCTGCCACTAAG |  |  |
|  | RP | TTCAGTAGGTTACCACACCATCCTG |  |  |
| All *cre* strains | Cre FP | TGCCAGGATCAGGGTTAAAGAT | 60°C | Cre + = 400 bp |
|  | Cre RP | AGCTTGCATGATCTCCGGTATT |  |  |
| *Pcdh15^n38/n38^*  (RT-PCR) | FP | TGGCCTTCATCATCATCCTCTGT | 69°C | WT = 559 bp |
|  | RV | TCAAGGCGCCTCCTCCACTC |  |  |
