# Supplementary material for: Role of Pcdh15 in the development of intrinsic polarity of inner ear hair cells
Source: PLoS Genet. 2025 Aug 13;21(8):e1011825. doi: 10.1371/journal.pgen.1011825 (PMC12370195; doi:10.1371/journal.pgen.1011825)
Supplement: S2 Table — (DOCX) [file pgen.1011825.s009.docx]

**S2 Table**

| **Primary antibodies used for immunofluorescence assays** | | | | |  |
| --- | --- | --- | --- | --- | --- |
| **Antibodies used against** | **Source** | **Catalogue No.** | **Fixation** | **Blocking buffer in 0.3% PBST (Tween-20)** |  |
|  |  |  |  |  |  |
| Acetylated tubulin | Proteintech | 66200-1-lg | 4% PFA, 4 hours, 4 ̊C | 10% GS + 1% BSA |  |
| ARL13B | Proteintech | 17711-1-AP | 4% PFA, 4 hours, 4 ̊C | 10% GS + 1% BSA |  |
| Daple | Bethyl Laboratories | A302-951A | 10% TCA, 1 hour, RT | 10% GS + 1% BSA |  |
| FLAG | Sigma | F7425 | 4% PFA, 4 hours, 4 ̊C | 10% GS + 1% BSA |  |
| G⍺i3 | Abcam | ab14246 | 4% PFA, 2 hours, 4 ̊C | 10% GS + 1% BSA |  |
| Girdin | R & D Systems | AF5345 | 10% TCA, 1 hour, RT | 5% BSA |  |
| HA | Abcam | ab9110 | 4% PFA, 4 hours, 4 ̊C | 10% GS + 1% BSA |  |
| GPSM2/LGN | Gift From Prof. Fumio Matsuzaki | | 4% PFA, 2 hours, 4 ̊C | 10% GS + 1% BSA |  |
| PCDH15 | R & D Systems | AF6729 | 4% PFA, 4 hours, 4 ̊C | 5% BSA |  |
| VANGL2 | R & D Systems | AF4815 | 4% PFA, 2 hours, 4 ̊C | 10% GS + 1% BSA |  |
| Pericentrin | Covance | PRB-432C | 4% PFA, 4 hours, 4 ̊C | 10% GS + 1% BSA |  |
| β-spectrin II | BD Biosciences | 612562 | 4% PFA, 4 hours, 4 ̊C | 10% GS + 1% BSA |  |
|  |  |  |  |  |  |
| **Primary antibodies used for western blotting** | | | | |  |
| **Antibodies used against** | **source** | **Catalogue No.** | **Dilution** | **Blocking buffer in 0.1% TBST (Tween-20)** |  |
|  |  |  |  |  |  |
| FLAG | Sigma | F3165 | 1:4000 | 5% BSA |  |
| HA | Sigma | H3663 | 1:5000 | 5% BSA |  |
| β-actin | BD Biosciences | 612656 | 1:5000 | 5% BSA |  |
