# Supplementary material for: Role of Pcdh15 in the development of intrinsic polarity of inner ear hair cells
Source: PLoS Genet. 2025 Aug 13;21(8):e1011825. doi: 10.1371/journal.pgen.1011825 (PMC12370195; doi:10.1371/journal.pgen.1011825)
Supplement: S3 Table — (DOCX) [file pgen.1011825.s010.docx]

**S3 Table**

**List of secondary antibodies used for immunofluorescence assays.**

| **Antibodies** | **source** | **Catalogue No.** |
| --- | --- | --- |
| Alexa Fluor 488 Phalloidin | Thermo Fisher Scientific | A-12379 |
| Alexa Fluor 568 Phalloidin | Thermo Fisher Scientific | A-12380 |
| Donkey anti-Mouse IgG Alexa Fluor 488 | Thermo Fisher Scientific | A-21202 |
| Donkey anti-Mouse IgG Alexa Fluor 594 | Thermo Fisher Scientific | A-21245 |
| Donkey anti-Sheep IgG Secondary Antibody, Alexa Fluor 488 | Thermo Fisher Scientific | A-11015 |
| Goat anti-chick IgG Secondary Antibody, Alexa Fluor 594 | Thermo Fisher Scientific | A-11042 |
| Goat anti-Mouse IgG Secondary Antibody, Alexa Fluor 488 | Thermo Fisher Scientific | A-11001 |
| Goat anti-Mouse IgG Secondary Antibody, Alexa Fluor 594 | Thermo Fisher Scientific | A-11032 |
| Goat anti-Mouse IgG Secondary Antibody, Alexa Fluor 647 | Thermo Fisher Scientific | A-21235 |
| Goat anti-Rabbit IgG Secondary Antibody, Alexa Fluor 488 | Thermo Fisher Scientific | A-11008 |
| Goat anti-Rabbit IgG Secondary Antibody, Alexa Fluor 647 | Thermo Fisher Scientific | A-21245 |
| Goat anti-Rat IgG Secondary Antibody, Alexa Fluor 488 | Thermo Fisher Scientific | A-11007 |
| Donkey anti-Mouse IgG Secondary Antibody, Alexa Fluor 405 | Thermo Fisher Scientific | A-48257 |
| Donkey anti-Rabbit IgG Secondary Antibody, Alexa Fluor 647 | Thermo Fisher Scientific | A-31573 |
