# Supplementary material for: Role of Pcdh15 in the development of intrinsic polarity of inner ear hair cells
Source: PLoS Genet. 2025 Aug 13;21(8):e1011825. doi: 10.1371/journal.pgen.1011825 (PMC12370195; doi:10.1371/journal.pgen.1011825)
Supplement: Attachment — Submitted filename: D-24-01537.docx [file pgen.1011825.s013.docx]

In this manuscript by Kaushik et al the contribution of protocadherin 15 (Pcdh15) towards stereociliary bundle development is evaluated using a unique conditional allele that produces a splicing-isoform specific knockout that can be converted to an epitope tagged and phosphorylation-deficient mutant. Using this line the authors expand upon prior characterizations of Pcdh15 and propose a model in which Pcd15-CD2 contributes to hair cell planar polarity. The KO has a striking impact on stereociliary bundle morphology in which the kinocilium is frequently separated from the stereocilia (kinicilium-stereocilia decoupling). As a result, the position of the kinocilium on the apical cell surface lacks the distinctive polarized localization to adjacent to the tallest stereocilia. In contrast, other aspects of hair cell intrinsic planar polarity and planar cell polarity are not disrupted in this mutant. For example, the localization of the intrinsic polarity factor Galpha i remains polarized with the protein enriched on the lateral edge of the apical hair cell surface. In addition, the rows of stereociliary bundles appear appropriately polarized and organized in rows of increasing height that are correctly positioned adjacent to Galpha i. Lastly, the polarized distribution of Core PCP proteins like Vangl2 is not disrupted in the Pdc15-Cd2 mutants. Altogether these results raise the question of how cell intrinsic planar polarity should be defined, and what phenotype criteria are needed to categorize it as disrupted. The intellectual merit of the manuscript would be improved if the authors discussed these questions and provided a clear argument as to why Pcd15-CD2 is contributing to intrinsic planar polarity as per the title rather than maintaining stereociliary bundle integrity by linking the kinocilium to the stereocilia.

Additional concerns

1) What is Pcdh15-CD2 and how is it different from Pdch15-CD1 and Pcdh15-CD-3? The introduction would benefit from the addition of an introduction to Pcdh15 alternative splicing and isoform function.
2) Figure 1: Since the kinocilium is decoupled from the stereocilia in the n38 mutants and the kinocilium and stereocilia seem to be differentially effected, the authors should make independent polarity measures for stereocilia polarization.
3) Line 244: Since the Preyer’s reflex is not applied to n38YF mice so it is not clear whether or not they can hear based on this assay. Without that result it is not accurate for this section of the manuscript to be titled “Tyrosine phosphorylation of the CD2 domain is not essential for Pcdh15 Function in mice” since the contribution phosphorylation towards hearing has not been tested.
4) Preyer’s reflex is not a robust or rigorous assay for auditory function and the authors should consider ABRs
5) Fig.7A: unlike Galpha I, the area of cell surface containing GPSM2 does not change in Pcdh15 KOs despite the colocalization of these proteins reported by others. This result is unexpected and should be discussed.

Minor concerns

1) It would be useful to report the frequency of circular bundles since they are not common in other figure panels and so that the may be compared to the frequency of other mutants like IFT88
2) Line 44: Hearing perception does not occur in the cochlea but rather in auditory cortex. This should be corrected.
3) Line 81: Since a hair cell only has one kinocilium this phenotype should be ‘kinocilium-stereocilia decoupling’
4) It would help your readers if the text stated that Pgk1-Cre was used to make a germline mutant particularly since Cre is more often used for cell or tissue-specific gene deletion
5) Figure 1 schematic: it is not obvious what parts of the protein are encoded by exon 38 or how alternative splicing could exchanges CD1 or CD3 for CD2
6) Fig8: The genotype nomenclature “ -/- “ that is used for the wild type Atoh1 allele is confusing since “ -/- “ typically indicates a null allele or knockout. Why not say Cre-negative or nothing at all? Similarly, the abbreviations ‘-ve’ and ‘+ve’ for positive and negative can be confusing. Why not just use HA-positive and HA-negative?
