# Supplementary material for: Role of Pcdh15 in the development of intrinsic polarity of inner ear hair cells
Source: PLoS Genet. 2025 Aug 13;21(8):e1011825. doi: 10.1371/journal.pgen.1011825 (PMC12370195; doi:10.1371/journal.pgen.1011825)
Supplement: Attachment — Submitted filename: Response to Reviewers.docx [file pgen.1011825.s015.docx]

Reviewer #1: In the current manuscript, Kaushik et al. have been studying the function of PCDH15 in mechanosensory hair cells. Previous studies have shown that PCDH15 is expressed in three isoforms, which differ in their cytoplasmic domains and are generated by alternative splicing. It had also been shown that one of these isoforms, PCDH15-CD2, is a component of kinociliary links that connect the longest stereocilia of hair cells to the kinocilium. Furthermore, in PCDH15-CD2 mutant mice, planar cell polarity (PCP) of hair cells is disrupted leading to misorientation of the stereocilia bundle in the apical hair cell surface. Finally, studies from the laboratory presenting the current study have provided evidence that the CD2 cytoplasmic domain of PCHD15 is phosphorylated on tyrosine residues. The authors thus wanted to address the mechanism by which PCDH15-CD2 and its tyrosine phosphorylation affect PCP.

To conduct their studies, the authors generated mutant mice lacking the PCDH15-CD2 isoform or carrying mutations in the tyrosine residues within CD2. They demonstrated that CD2 tyrosine phosphorylation is not required for PCDH15-CD2 function in PCP, but they confirm earlier findings that PCP is disrupted in PCDH15-CD2 mutant mice. They then use immunolocalization studies for PCDH15-CD2 and other genes linked to PCP signaling such as GPSM2 and Gai to study effects of the PCDH15-CD2 mutation. They also generate PCDH15-CD2 and GPMS2 compound mutant mice to define potential genetic interactions between PCDH15-CD2 and genes linked to PCP signaling. They conclude that PCDH15-CD2 affects PCP independent from its function in the formation of kinociliary links.

Unfortunately, while the authors have done a lot of work, I do not find that the experiments support the conclusion put forward. Substantial more work would be necessary to establish what might be an interesting idea. There are also several technical limitations to the study:

1.The authors argue that PCDH15-CD2 is expressed at the base of kinocilia prior to the formation of kinociliary links. The immunohistochmical data are not convincing (Fig. 3). They use PCDH15-CD2-HA mice, which express an epitope tagged PCDH15-CD2 to reach the conclusions. They do not show non-HA controls. The PCDH15-CD2-HA mice carry several mutations in CD2, which could affect expression or localization. Also, more convincing pictures of HA staining would be required such as co-staining with markers for the base of the kinocilium and sagittal views of sectioned hair cell. There are also PCDH15 antibodies available from several laboratories to confirm the findings. Publications from the Barr-Gillespie laboratory show what kind of resolution can now be obtained to visualize subcellular distribution of proteins in hair cells. Finally, the staining data should be quantified.

We thank the reviewer for highlighting this point.

To address their concern regarding the specificity and reproducibility of the immunohistochemical data, we confirmed our results using three independent HA-specific antibodies (catalog numbers: ab9110, 12CA5, H3663). All antibodies yielded identical expression patterns. We also attempted staining with the PCDH15-CD2 antibody (generated by Müller’s group), but were unable to obtain consistent or reliable data with this reagent.

*Fig. 1: Modified PCDH15-CD2-HA (green) is expressed in the stereocilia (magenta) of Pcdh15^n38YF/n38YF^ hair cells (P0, mid). Apart from the main HA antibody (ab9110) used, this expression is confirmed with the help of two other HA-specific antibodies (12CA5 and H3663).*

To validate the specificity of the HA signal, we performed staining in wild-type littermates that do not express the Pcdh15-CD2-HA construct. These negative controls showed no HA signal (see Fig. 2C), confirming the specificity of our staining. We have included the non-HA control for E16.5 stage as well.

*Fig. 2: Non-HA controls for E16.5 organ of Corti, utricle and cristae.*

As the reviewer correctly notes, PCDH15-CD2-HA mice carry six Y-to-F mutations in exon 38 of the CD2 domain. While this raised the possibility of altered localisation, however, we observed robust and consistent localisation of PCDH15-CD2 in the stereociliary bundles of hair cells in both the organ of Corti and the vestibular sensory epithelium. This localisation pattern was stable across developmental stages and aligns with previous reports (Ahmed et al., 2006; Webb et al., 2011; Pepermans et al., 2014).

We agree that demonstrating the spatial relationship between PCDH15-CD2-HA and markers for the base of the kinocilium is important. In response, we performed staining of PCDH15-CD2-HA with Acetylated tubulin and centrioles marker (ODF-2) on the sagittal sections of the mouse organ of Corti. Unfortunately we could not capture the centrioles attached to the kinocilium in these samples.

Alternatively, we co-immunostained for acetylated tubulin, a marker that labels the full length of the kinocilium including its transition zone (Najarro et al., 2020). We analysed these data in 3D using Imaris software, and have now included representative images and quantification in the revised manuscript. Further, we validated HA staining by co-staining with a PCDH15-specific antibody; these results are also presented in the revised version (see Fig. S2B).

Lastly, we acknowledge that the Barr-Gillespie lab has produced excellent hair bundle images with higher resolution than ours. However, it is important to note that their imaging is largely from postnatal stages when stereocilia are better developed and more spatially separated, particularly in inner hair cells. In contrast, our imaging focuses on E15.5–E16.5 stages of cochlear development, when hair bundle structures are still compact and more challenging to resolve.

We hope these additional data and clarifications address the reviewer’s concerns.

2.The authors stain hair cells around E16.5-E18.5 to support the conclusion that labeling at the base of kinocilia is observed prior to the presence of kinociliar links. However, kinociliary links are already present at this age (even in the publication from the Richardson lab they cite, which is not the most convincing example, kinociliar links are present at this age, especially at the base and top of stereocilia). If the authors want to make this point, they would have to show that unlike what is published, they cannot find kinociliary links at an age when they observe PCP defects.

We thank the reviewer for pointing this out and appreciate the opportunity to clarify our observations.

In the manuscript, we show the expression of PCDH15-CD2 in the kinocilia of outer hair cells (OHCs) located in the apical region of the E16.5 organ of Corti (Fig. 3B). At this developmental stage and location, the apical OHCs are still immature: microvilli have not yet fully differentiated into stereocilia, and the kinocilium has not undergone its characteristic off-center migration (Fig. A, below, and Chakravarthy et al., 2024).

*Fig. 3: SEM imaging of the mouse E16.5 organ of Corti showing different stages of hair bundle development.*

To our knowledge, no published reports have demonstrated the presence of kinocilial links in apical OHCs at E16.5, supporting the novelty of our observation. To investigate this further, we performed scanning electron microscopy (SEM) on E16.5 cochlear tissue. In Pcdh15-n38 mutant mice, planar cell polarity (PCP) defects are first evident in OHCs at the mid-cochlear region around E16.5. Even in these regions where PCP phenotypes are apparent, we did not observe kinocilial links in OHCs (highlighted in section B), further suggesting that kinocilial link formation may occur later in development or be more prominent in IHCs (section C).

3.The authors show that polar Gai localization is affected in PCDH15-CD2 mice (Fig. 5A-C). This is interesting but may not be unexpected since PCP is affected in the mutants. Thus, the localization of PCP components might be perturbed. The problem is that we do not know what is cause and what is consequence of what. No experiment directly addresses a mechanistic link between PCDH15-CD2 and Gai.

While the altered polar localisation of Gαi observed in the PCDH15-CD2 mutant (Fig. 5A–C) could reflect a broader polarity defect, the fact that markers of PCP are unperturbed (Fig. S1D) suggests a role of Pcdh15-CD2 in intrinsic polarity. However, establishing a causal mechanistic link between PCDH15-CD2 and Gαi localisation will require further investigation. However, we believe our findings provide insight into the role of PCDH15-CD2 in intrinsic planar polarity of hair cells, distinct from classical tissue-level PCP.

Gαi is a well-established marker of intrinsic apical polarity, associated with the asymmetric distribution of apical membrane domains in developing hair cells (Ezan et al., 2013; Tarchini et al., 2013). Its polar localisation does not depend on tissue-level PCP cues, but rather reflects the intrinsic organisation of the cytoskeleton and apical specialisations, including the kinocilium and stereociliary bundle. Given that PCDH15-CD2 is required for maintaining hair bundle integrity, and that the Pcdh15n38/n38 mutants show hallmarks of disrupted intrinsic polarity (e.g. dissociation of the kinocilium from the bundle), we interpret the Gαi mislocalisation as a readout of disrupted intrinsic polarity, rather than a secondary consequence of PCP disruption. While we do not show a direct molecular interaction between PCDH15-CD2 and Gαi, our results support a model where PCDH15-CD2 influences apical membrane organisation, potentially by influencing the cytoskeletal or membrane asymmetries necessary for Gαi polarisation.

We have revised the discussion to clarify that our data establishes a correlative link between PCDH15-CD2 function and intrinsic polarity defects, as evidenced by Gαi mislocalisation, but do not claim a direct mechanistic connection. We appreciate the reviewer’s suggestion and have used it to sharpen the interpretation of our findings.

4.The authors suggest that pericentrin localization may be affected in PCDH15-CD2 mutant mice (Fig. 4G,H). The images are not convincing and would need quantification. How do we know what is the mother/daughter centriole.

We thank the reviewer for this suggestion. Although we think that the highlighted image shows misorientation of centrioles in Pcdh15^n38/n38^ mice along with the mispositioned kinocilia, we have quantified this, and provided the segmented images. We have provided a single optical section of the image showing connection between the kinocilium (Acetylated tubulin) and centrioles (pericentrin) where two dots (mother and daughter centrioles) are visible in Pcdh15-CD2 mutant hair cells as compared to their littermate controls. This suggests that in Pcdh15-CD2 mutant hair cells, the position of the centrioles is perturbed in z-direction with respect to each other.

Misorientation of centrioles with mispositioned kinocilia is reported in hair cells of other PCP mutants (Sipe and Lu, 2011; Siletti et al., 2017). Additionally, Webb et al., 2011 has reported similar mispositioning of centrioles using pericentrin staining in Pcdh15-△CD2 mice hair cells. We have identified the mother centriole if it is attached to the kinocilium and daughter centriole if not attached.

We thank the reviewer for suggesting the quantification which we have included in the revised manuscript.

5.Intercrosses between PCDH15-CD2 and GPMS2 mutant mice suggest that the two genes genetically interact. The effect is particularly striking for the vestibule, where PCDH15-CD2/GPMS2 mutant mice have a far more pronounced hyperactivity defect than single mutants. However, vestibular hair bundles are not investigated. It should also be noted that there is a profound difference in the kinocilia of cochlear and vestibular hair cells. In the cochlea kinocilia in hair cells are transiently present during development, but in the vestibule they are maintained into adulthood where they are thought to be important for stereocilia stimulation by mechanical force. Thus, the vestibular impairment could indicate a functional defect in hair cells that have to do with coupling of the hair bundle to the overlying matrix to enable efficient force sensing. Or the stereocilia might have degenerated. Or any of several other possibilities. This would likely need a separate detail study to define mechanisms.

We thank the reviewer for pointing this out and appreciate the opportunity to expand our observations on the intercrosses between PCDH15-CD2 and GPSM2 mutant mice.

To investigate the vestibular impairment responsible for the circling behavior, first we imaged the hair bundles of utricle, saccule and crista with the help of SEM. In comparison to control hair cells, stereocilia bundles were shorter in the utricle, saccule and crista of GPSM2 mutant mice as well as the double mutant mice. These bundles were comparable in Pcdh15-CD2 mutant mice and the control mice hair cells.

To understand the basis of circling behavior especially in the double mutant mice and not in GPSM2 mice, we compared the hair bundle morphology of crista between these mice. We observed that the stereociliary length defects were comparable in the utricle and saccule of the GPSM2 mutant mice and the double mutant mice, but there was some gradation preserved in the stereocilia bundles of the GPSM2 mutant crista hair cells which was lost in the double mutant mice. Although we do not know if this defect in the stereocilia bundle is enough to explain the circling behavior in the double mutant mice, it suggests that the morphological defects are pronounced in these mice compared to Pcdh15-CD2 or GPSM2 mutant mice.

Mauriac et al., (2017) have observed circling behavior in GPSM2 cKO mutant mice where they have deleted the exon 5-7 of GPSM2 gene in the inner ear. They have also reported a reduction in the stereocilia length of the GPSM2 mutant mice as a possible reason for the observed behavior. In contrast, the GPSM2 mutant mice (GPSM2 delta C mice) with C-terminal GoLoco motifs deleted (last three exons deleted), reported by Bhonker et al., 2018 (made by Konno et al., 2008) do not show any vestibular defects. Similarly, the GPSM2 allele in this study, (in which exon 3-15 of GPSM2 coding region is deleted, Fujita et al., 2020) does not show circling behaviour, although the stereocilia are shorter. We do not understand this differential phenotype between these GPSM2 alleles.

We also asked if we observe polarity defects in crista of these mice. We found a significant increase in the misorientation of hair cells in both Pcdh15-CD2 and GPSM2 mutants as compared to their littermate controls. In the absence of circling behavior in these mice, this data suggests that the difference in hair cell orientation between these mice is not enough to develop this phenotype. However, hair cell misorientation is significantly increased in the double mutant mice, but again, we do not know if this enhanced misorientation of hair cells is enough to elicit the circling behavior in these mice.

Reviewer #2: In this manuscript by Kaushik et al the contribution of protocadherin 15 (Pcdh15) towards stereociliary bundle development is evaluated using a unique conditional allele that produces a splicing-isoform specific knockout that can be converted to an epitope tagged and phosphorylation-deficient mutant. Using this line the authors expand upon prior characterizations of Pcdh15 and propose a model in which Pcd15-CD2 contributes to hair cell planar polarity. The KO has a striking impact on stereociliary bundle morphology in which the kinocilium is frequently separated from the stereocilia (kinicilium-stereocilia decoupling). As a result, the position of the kinocilium on the apical cell surface lacks the distinctive polarized localization to adjacent to the tallest stereocilia. In contrast, other aspects of hair cell intrinsic planar polarity and planar cell polarity are not disrupted in this mutant. For example, the localization of the intrinsic polarity factor Galpha i remains polarized with the protein enriched on the lateral edge of the apical hair cell surface. In addition, the rows of stereociliary bundles appear appropriately polarized and organized in rows of increasing height that are correctly positioned adjacent to Galpha i. Lastly, the polarized distribution of Core PCP proteins like Vangl2 is not disrupted in the Pdc15-Cd2 mutants. Altogether these results raise the question of how cell intrinsic planar polarity should be defined, and what phenotype criteria are needed to categorize it as disrupted. The intellectual merit of the manuscript would be improved if the authors discussed these questions and provided a clear argument as to why Pcd15-CD2 is contributing to intrinsic planar polarity as per the title rather than maintaining stereociliary bundle integrity by linking the kinocilium to the stereocilia.

We thank the reviewer for their thoughtful summary of our work and for highlighting a key issue in the field. We fully agree that some confusion persists in the literature regarding the categorisation of planar polarity defects in the inner ear, particularly due to conflation of intrinsic planar polarity at the level of individual hair cells and tissue-level planar cell polarity (PCP) involving coordinated orientation across the epithelium.

As the reviewer rightly points out, these are distinct processes, and this distinction is critical for correct interpretation of phenotypes. Intrinsic planar polarity refers to the asymmetric cytoskeletal organisation within individual hair cells and is characterised by the graded array of stereocilia anchored by a kinocilium at the vertex, and supported basally by a cuticular plate and underlying microtubules. This process is independent of the global tissue axis and has been defined in multiple studies (Tarchini et al., 2013; Siletti et al., 2017; Tarchini & Lu, 2020; Deans, 2021; Tarchini, 2021).

In contrast, tissue-level PCP describes the uniform orientation of hair cells across the epithelium and is regulated by core PCP signalling components. The phenotypic criteria for disrupted intrinsic polarity, as established by Tarchini (2021), include dysmorphic cytoskeletal organisation within the hair bundle, such as the detachment of the kinocilium from the stereociliary V-structure, a phenotype observed in our Pcdh15n38/n38 mutants.

We agree with the reviewer that PCDH15-CD2 is crucial for regulating the apical cytoskeletal asymmetry in HC, as well as maintaining hair bundle integrity, and therefore its function falls under the domain of intrinsic polarity regulation. We have clarified this framework in the revised Discussion to better distinguish between intrinsic and tissue-level planar polarity and to accurately contextualise our findings.

We thank the reviewer for prompting this clarification.

Additional concerns
1)What is Pcdh15-CD2 and how is it different from Pdch15-CD1 and Pcdh15-CD-3? The introduction would benefit from the addition of an introduction to Pcdh15 alternative splicing and isoform function.

We thank the reviewer for pointing this out. We have added the details about different isoforms of Pcdh15 in the introduction.

2)Figure 1: Since the kinocilium is decoupled from the stereocilia in the n38 mutants and the kinocilium and stereocilia seem to be differentially effected, the authors should make independent polarity measures for stereocilia polarization.

We thank the reviewer for this helpful suggestion and agree that it is important to analyse stereocilia bundle orientation independently of other polarity readouts. In response, we measured the orientation of individual stereociliary bundles relative to the tissue axis and quantified the angular deviation as a readout of planar polarisation. Our analysis revealed a significant increase in bundle misorientation in Pcdh15n38/n38 mutants compared to littermate controls. Interestingly, the defects were more pronounced in the OHC1 and OHC2 rows, while OHC3 displayed comparatively milder deviations. We speculate that this may reflect the ongoing maturation and refinement of OHC3 polarity in the wild-type cochlea at the developmental stage examined.

These new data have been included in the revised manuscript as Figure S1E, and the accompanying text has been updated accordingly.

3)Line 244: Since the Preyer’s reflex is not applied to n38YF mice so it is not clear whether or not they can hear based on this assay. Without that result it is not accurate for this section of the manuscript to be titled “Tyrosine phosphorylation of the CD2 domain is not essential for Pcdh15 Function in mice” since the contribution phosphorylation towards hearing has not been tested.

We thank the reviewer for pointing this out and we agree with the reviewer's comment that the contribution of Pcdh15-CD2 phosphorylation towards hearing has not been tested. We have measured the Preyer’s reflex response in the n38YF mice and found the startle response comparable to wild-type mice. We have included the data in the revised manuscript. This is now shown in Fig. S2G.

4)Preyer’s reflex is not a robust or rigorous assay for auditory function and the authors should consider ABRs

We fully agree and acknowledge the limitations of Preyer’s reflex in terms of sensitivity and quantification.

However, we note that the auditory deficit in Pcdh15-CD2 mutant mice has already been well established through detailed auditory brainstem response (ABR) recordings in previous studies (Webb et al., 2011; Pepermans et al., 2014). Our aim was not to re-characterize this known phenotype, but rather to verify the expected deafness phenotype using a method that is simpler and cost-effective. We have included the outcome of the Preyer’s reflex test and an explanation of the scoring method used in the revised manuscript. These are meant to confirm the phenotype qualitatively and are not intended to replace more quantitative auditory assays.

5)Fig.7A: unlike Galpha I, the area of cell surface containing GPSM2 does not change in Pcdh15 KOs despite the colocalization of these proteins reported by others. This result is unexpected and should be discussed.

We apologise to the reviewer for our oversight. Gpsm2 **is** altered, similar to Galphai - the expression is expanded towards the medial side of the HC. We performed an analysis for this expansion, similar to Galphai. We have included this analysis data in the revised manuscript (Fig. S4).

Minor concerns
1)It would be useful to report the frequency of circular bundles since they are not common in other figure panels and so that the may be compared to the frequency of other mutants like IFT88

The frequency of circular bundles is <1% in Pcdh15-CD2 mutant mice OC, while this is not statistically significant, we have mentioned this in the text.

2)Line 44: Hearing perception does not occur in the cochlea but rather in auditory cortex. This should be corrected.

We thank the reviewer for this: We have changed this in the revised manuscript.

3)Line 81: Since a hair cell only has one kinocilium this phenotype should be ‘kinocilium-stereocilia decoupling’

We agree with the reviewer and we have corrected it in the manuscript.

4)It would help your readers if the text stated that Pgk1-Cre was used to make a germline mutant particularly since Cre is more often used for cell or tissue-specific gene deletion

We thank the reviewer for this suggestion and we have changed this in the revised manuscript.

5)Figure 1 schematic: it is not obvious what parts of the protein are encoded by exon 38 or how alternative splicing could exchanges CD1 or CD3 for CD2

We thank the reviewer for pointing this out and we have made this change in the revised manuscript.

6)Fig8: The genotype nomenclature “ -/- “ that is used for the wild type Atoh1 allele is confusing since “ -/- “ typically indicates a null allele or knockout. Why not say Cre-negative or nothing at all? Similarly, the abbreviations ‘-ve’ and ‘+ve’ for positive and negative can be confusing. Why not just use HA-positive and HA-negative?

We thank the reviewer for this suggestion and we have made these changes in the revised manuscript.

Reviewer #3: In this manuscript, Kaushik et al. aimed to understand the role of Pcdh15 in planar cell polarity (PCP) establishment of hair cells in the developing cochlea. They reported a Pcdh15-n38 knock-in mouse which was designed to tag the endogenous Pcdh15-CD2 isoform, but found that the inserted FLAG tag was undetectable, and the mice were phenotypically similar to Pcdh15-CD2 knockouts in terms of PCP and centriolar defects. Upon Cre-mediated recombination, their allele did express an HA-tagged, phospho-dead CD2 domain, which partly rescued PCP defects. These results and the immunodetection of CD2 using the HA tag suggested an early role of Pcdh15 in PCP establishment independent of its interaction with the kinocilium. Furthermore, the authors generated compound mutants with Pcdh15-n38 and Gpsm2, showing genetically synergistic effects in PCP. These results are original and important to further understanding the mechanism of PCP establishment in hair cells. The paper is overall technically sound, but some conclusions are not fully supported by the data, and some interpretations do not come through (detailed below). The writing can be improved to be more understandable for general audience, as some technical terms specific to the hair cell biology were not defined when they were first used. Some essential information such as the sample size, definition of axes in the figure, and abbreviations in the figure are missing.

We thank the reviewer for summarizing our work nicely and for the valuable suggestions. We have incorporated the suggested changes in the revised manuscript that include defining the terms specific to hair cell biology, sample size, figure axes and abbreviations.

1. Missing definition: CD2, bare zone, ve (Fig. 8), arrows in the figures.

We thank the reviewer for pointing this out and we have defined the CD2 isoform of Pcdh15 with other isoforms in the introduction of the revised manuscript. We have defined the bare zone in the revised manuscript. We have corrected the ‘ve’ in (Fig. 8) and included the arrows in the figures (wherever required) in the revised manuscript.

2. Western blot in Fig. 1C should have a loading control. Also, the authors should give some explanations/speculations about why the n38 allele did not express before Cre recombination. Was is truly equivalent to CD2 knockout at the molecular level? Is it possible to characterize the transcript at the RNA level?

We thank the reviewer for this important point. We agree that the inclusion of a loading control is important. We have now repeated the experiment and included appropriate loading controls in the revised figure. This updated data is now included in the revised manuscript (Fig. 1D, S1B).

Regarding the lack of detectable protein from the n38 allele, we investigated whether this was due to a transcriptional or translational issue. To address this, we amplified the Pcdh15-CD2 transcript spanning exon 31 to exon 38 from organ of Corti tissue in Pcdh15n38/n38 mice and found mRNA levels to be comparable to wild-type littermates (Figure S1C). We further validated the integrity of the amplified transcript by sequencing, which confirmed the expected nucleotide sequence, including the presence of the engineered mutations. These results suggest that the n38 allele is transcribed normally but may not be efficiently translated. One plausible explanation could be related to post-transcriptional regulation, particularly involving the poly(A) tail. The distance between the coding region and the polyadenylation site, or the presence of an alternative polyadenylation site (in the YF segment), could potentially impair translation efficiency. As discussed in the revised manuscript, the poly(A) tail has been implicated in multiple aspects of translational control, including: (1) Facilitating nuclear export of the transcript, (2) Promoting translation initiation via the closed-loop model involving interactions between the 5’ cap and the poly(A) tail, and (3) Recruiting poly(A)-binding protein (PABPC), which enhances translation efficiency and protects mRNA from degradation (Passmore & Coller, 2021).

While our current study does not directly test these mechanisms, we have included this speculation in the revised discussion.

3. In Fig. 4GH, it is unclear what "right angles" mean. Quantification of the angles would make more sense.

We thank the reviewer for pointing this out and we apologize for assuming the organisation of the mother-daughter centrioles as 90 degree (hence right angles). Pericentrin staining in control hair cells is visible as a single dot when viewed apically. We assumed that this is because the centrioles are on top of each other (arranged orthogonally, making a T). In two-dimensional images of Pcdh15-n38 mutant hair cells, we could see both the centrioles separated. The segmented image shows misorientation of centrioles in Pcdh15^n38/n38^ mice, we have quantified this (Fig. 4G-H). We have provided a single optical section of the image showing connection between the kinocilium (Acetylated tubulin) and centrioles (pericentrin) where two dots (mother and daughter centrioles) are visible in Pcdh15-n38 mutant hair cells as compared to their littermate controls (Fig. 4I, S3). This suggests that in Pcdh15-n38 mutant hair cells, the position of the centrioles is perturbed in z-direction with respect to each other. This observation is in agreement to the already reported Pcdh15-CD2 mutant mice from Muller’s group (Webb et al., 2011).

4. Line 337, why is the interaction between Pcdh15 and Gpsm2 likely to be downstream? A synergistic effect could happen with a common upstream regulator. Line 414 said their localizations were independent, which is contradictory to Fig. 5, where the n38 mutation changed Gai localization.

We apologise to the reviewer for our oversight. Gpsm2 **is** altered, similar to Galphai - the expression is expanded towards the medial side of the HC. We performed an analysis for this expansion, similar to Galphai. We have included this analysis data in the revised manuscript. The reviewer is correct - we cannot say whether the interaction is downstream or if a synergistic effect could happen with a common upstream regulator. We have removed this contention.

5. In Fig. 8D, it seems that the HA-negative cells also had a significant rescue of n38 in kinocilia deviation, which needs explanation.

We thank the reviewer for raising this point. We agree with the reviewer about the significant rescue of HA-negative cells in the n38 rescue mice. Although we do not have any direct explanation for this observation, we think of it as a classical case of domineering non autonomy which has been well reported in the inner ear system (Stoller et al., 2018; Prakash et al., 2025). We think that similar to domineering non autonomous phenotype observed during the PCP signalling disruption leading to polarity defects in neighboring wild type cells, the recovery in kinocilia deviation in HA-negative cells might be happening. This is hypothesised to be happening because of local intercellular signalling operating in hair cells of the organ of Corti.
