# Supplementary material for: Role of Pcdh15 in the development of intrinsic polarity of inner ear hair cells
Source: PLoS Genet. 2025 Aug 13;21(8):e1011825. doi: 10.1371/journal.pgen.1011825 (PMC12370195; doi:10.1371/journal.pgen.1011825)
Supplement: Attachment — Submitted filename: Rebuttal 2.docx [file pgen.1011825.s016.docx]

*Reviewer #1: The authors have substantially revised the manuscript. While the data are interesting, I do not find that the data support the conclusion that “Pcdh15-CD2 couples kinocilia and stereocilia development through a mechanism independent of kinocilial links” (abstract). They also conclude that Pcdh15-CD2 is linked to Gai/Gpsm2 signaling, which in my opinion is not clearly shown.

Confirmative findings:
Using new mouse models, the authors confirm earlier findings that the Pcdh15-CD2 isoform is expressed in the kinocilium of hair cells and required for coupling of the kinocilium to stereocilia. They also confirm that hair cells of Pcdh15-CD2 mutant mice have a defect in the intrinsic polarity as revealed in the morphology of stereocilia bundles.

New findings:
1.Phosphorylation of Pcdh15-CD2 is not crucial for the development of a polar hair bundle
2.Localization of Gai is perturbed in Pcdh15-CD2 mutant mice consistent with a defect in intrinsic polarity
3. Pcdh15-CD2 mutations seem to genetically interact with Gpsm2 mutations in both the cochlea and vestibule (but see comment below).

Major critique points:
1.I do not see convincing evidence that the polarity defects cannot be explained by defects in the coupling of the stereocilia to the kinocilium via kinociliar links. The authors argue that Pcdh15-CD2 is expressed prior to the formation of kinociliary links and that polarity defects materialize prior to the presence of kinociliary links. To me the supporting data are not of sufficient quality and resolution to reach that conclusion.*

Intrinsic polarity involves three distinct phases of kinocilium movement (Tarchini et al., 2013): (1) initial off-centre migration toward the lateral edge of the hair cell, (2) positioning within a crescent marked by Gpsm2/Gαi expression, and (3) final relocation of the maturing hair bundle toward the cell centroid as the bare zone forms.

Our data indicate that the defect in intrinsic polarity in Pcdh15-CD2 mutants arises from a failure in step 2, specifically, the inability of the kinocilium to position itself at the centre of the Gpsm2/Gαi crescent. This step, like hair cell differentiation, proceeds as a wave from the base to the apex of the cochlea and occurs between E16.5 and E17.5 (Tarchini et al., 2013).

It is difficult to reconcile this process with a mechanism based solely on adhesion between the kinocilium and the tallest stereocilium, given that these structures are spatially distant at this stage. This is even more pronounced in our rescue experiments; it is difficult to envision how the kinocilium would reattach to the stereocilia through adhesion alone (that too over the large gap developed in Pcdh15-n38 mutant HC) if that were the exclusive mechanism involved. However, to clarify the timing of kinociliary link formation, we consulted Dr. Peter Barr-Gillespie, who confirmed that these links are not present in outer hair cells (OHCs) at E16.5. As the reviewer notes, kinocilial links begin to appear in basal OHCs around E17.5. We observe clear defects in kinocilium positioning as early as E16.5, prior to the emergence of kinocilial links.

Taken together, these findings strongly support the conclusion that PCDH15-CD2 plays a distinct role in regulating intrinsic polarity, independent of its function in kinociliary link formation. We have clarified this interpretation in the revised Discussion.

*2.The fact that Gai is mis-localized in Pcdh15-CD2 mutants could be a consequence of polarity defects without a direct mechanistic link.*

We observe a medial-ward expansion in the Gai expression domain in mutants of Pcdh15-CD2. This is similar to the medial-ward shift seen in mutants that are involved in cilia formation (Mkks: Ezan et al., 2013; Bbs8: May-Simera et al., 2015; and Ift88: Bhonker et al., 2016). These led us to believe that the mislocalisation is due to a perturbation in the structure of the kinocilia, although establishing a direct mechanistic link between PCDH15-CD2 and Gαi localisation will require further study. Our data provide new insights into the function of PCDH15-CD2 in regulating intrinsic planar polarity, distinct from classical tissue-level PCP pathways. While we do not demonstrate a direct molecular interaction between PCDH15-CD2 and Gαi, our results support a model in which PCDH15-CD2 contributes to apical membrane organisation, potentially by maintaining the cytoskeletal and membrane asymmetries required for Gαi polarisation. We have revised the Discussion to clarify that our findings establish a correlative link between PCDH15-CD2 function and intrinsic polarity, without implying a direct mechanistic connection.

We thank the reviewer for this important point, which helped sharpen our interpretation and improve the clarity of the revised text.

*3.The genetic interaction data are interesting but also do not provide direct evidence of a mechanistic link. Weakening two molecular pathways that run in parallel (e.g.: kinocilia-stereocilia cohesion via Pcdh15-CD2, and molecular pathways regulating the polarity of the cytoskeleton) could explain stronger phenotypes. In addition, there is an issue with the quantification of the genetic interaction data. Fig. 6E lacks the proper control, comparison of double mutants to Pcdh15-n38/n38 mutants. Pcdh15-n38/n38 mutants are shown in Fig. 1H and it seems that kinocilia mislocalization is similarly affected in Pcdh15-n38/n38 mutant mice (Fig. 1H) compared to Pcdh15-n38/n38;Gspm2-/- mice (Fig. 6E). Perhaps the effect is slightly enhanced but since there is no direct statistical comparison, it is hard to be sure. Maybe a stronger phenotype shows up for fragmentation of hair bundles in double mutants (Fig. 6F), but this has not been quantified for Pcdh15-n38/n38 mutants. Likewise, the proper control (Pcdh15-n38/n38 mutant mice) is lacking for the analysis of the vestibular phenotype. Of note, it has previously been shown that Pcdh15-CD2 mutant mice have a vestibular defect.*

We agree with the reviewer that the genetic interaction data do not imply a direct mechanistic link. However, we would like to address several of the reviewer’s other comments regarding the interpretation of the double mutant phenotypes.

First, we compared the double mutant to the Pcdh15-CD2-null on a Gpsm2 heterozygous background, which we consider the more appropriate control for this genetic context. Regarding kinocilium positioning, the Pcdh15-CD2-null (Fig. 6B) does not exhibit a more severe polarity defect than the double mutant (Fig. 6D), as we had shown with quantitative analysis in Fig. 6E.

Second, fragmentation is enhanced in the double mutant, a point confirmed by statistical comparisons against the appropriate single-mutant controls, as presented in Fig. 6F.

Finally, a vestibular defect for Pcdh15-CD2 has **not** previously been shown. Webb et al., 2011 performed extensive tests on the vestibular function in their mutant and did not identify any vestibular defects, and similar conclusions were reached by Pepermans et al. (2014). We noted that the staircase of the hair bundle of cristae is lost in double mutants, but not in either of the signal mutants. We suggested (in the discussion) that this may underlie the circling behaviour, although the mechanism behind this cristae-specific defect is not known.

*Additional points:
1.Introduction: “hearing is sensed “ should be correcte. We do not sense hearing, we sense sound.*

We thank the reviewer for bringing this to our attention. We have corrected it in the revised manuscript.

*2.FM-143 uptake to study mechanotransduction: controls a lacking to show that uptake is via mechanotransduction channels and not by endocytosis. A control experiments should be performed: FM-143 uptake in the presence of channel blockers.*

FM1-43 dye is a well-established tool for assessing whether mechanotransduction (MET) channels are open. Gale et al. (2001) demonstrated that FM1-43 uptake occurs through MET channels and not via endocytosis, directly addressing the concern raised by the reviewer. Furthermore, Meyers et al. (2003) showed that dye uptake is significantly reduced in the presence of the MET channel blocker gadolinium—the very control suggested by the reviewer. Given this extensive prior validation, and the fact that FM1-43 has since been routinely used in the field to assess MET channel function, we do not believe that repeating this experiment would add any new information to our study.

*3.The authors conclude that the hearing defects (Preyers reflex) cannot be explained by a defect in mechanotransduction. They cannot conclude this. The FM-143 experiments were carried early postnatally, the hearing tests at P60. By that age, mechanotransduction could be affected.*

We agree with the reviewer and apologise for the lack of clarity in our original statement. To clarify, we fully concur that mechanotransduction is likely perturbed in adult mice, as opposed to the early postnatal stage. This would be consistent with previously reported phenotypes in Pcdh15-CD2 mutant models.

*4.The authors should measure ABRs, maybe at P21 and P60. Preyers reflex is insufficient to reveal if there are significant defects in auditory thresholds (maybe the phosphorylation mutants have a hearing defect not revealed by the crude assay).*

We thank the reviewer for this suggestion. At present, we do not have access to an ABR setup within our institute, and to our knowledge, such a facility for mice is not available in India. As a result, we used the simpler and more accessible Preyer’s reflex (click test) as a functional readout. We fully acknowledge the limitations of this method in terms of sensitivity and quantitative resolution, and these are explicitly noted in the Discussion.

*5.In Fig.3A, dots for HA staining are all over the tissue, sometimes with multiple dots per cells. Staining seems to be present in hair cells and supporting cells. Is this correct? Also, why not co-stain in Fig. 3A with kinociliary markers to solidify the conclusion that the staining is in kinocilia. And show controls, in parallel with mice not expression an HA-tagged protein to exclude background. I appreciate that staining controls are shown in the response letter, but they should be part of the main figure.*

We thank the reviewer for this comment. We agree that while HA antibody background could be a concern, we only detected this in cochlea from Pcdh15n38YF mice (where Pcdh15-CD2 is tagged with HA). We have included the co-staining with ciliary markers at E15.5, to support kinociliary localisation. In line with the reviewer’s suggestion, we have now included the relevant staining controls in the supplementary figure (Fig. S2).

*Reviewer #2: While the authors have made selective improvements to the manuscript, they have left my two primary concerns unaddressed:

1. What constitutes intrinsic polarity, and what phenotype criteria must be met to categorize it as disrupted?*

Intrinsic planar polarity refers to the asymmetric cytoskeletal organisation within individual hair cells and is characterised by the graded array of stereocilia anchored by a kinocilium at the vertex. This process is independent of the global tissue axis and has been described in multiple studies (Tarchini et al., 2013; Siletti et al., 2017; Tarchini & Lu, 2020; Deans, 2021; Tarchini, 2021). According to the criteria established by Tarchini (2021), disrupted intrinsic polarity is defined by aberrant cytoskeletal organisation within the hair bundle, most notably, detachment of the kinocilium from the stereociliary structure. We apply this to classify the phenotype observed in our Pcdh15n38/n38 mutants as a defect in intrinsic planar polarity.

*2. Why should Pcd15-CD2 be interpreted as a contributor to intrinsic planar polarity—as the title implies—rather than as a mediator of stereociliary bundle cohesion via kinocilium–stereocilia linkage?*

*I raised these points because I remain unconvinced that Pcd15-CD2 plays a direct role in establishing intrinsic hair cell planar polarity. That said, the authors should be given room to explain their rationale and clarify why they believe Pcd15-CD2 is not regulating kinocilium–stereocilium adhesion, with only indirect effects on cell orientation. They have not done so.*

We thank the reviewer for being open to the possibility that PCDH15-CD2 may have additional functions beyond its established role in kinociliary link formation.

To clarify, we do not suggest that PCDH15-CD2 is uninvolved in mediating kinocilium–stereocilia adhesion. Rather, we propose that it also plays an earlier, previously unrecognised role in intrinsic polarity establishment during the E15.5–E17.5 window. Intrinsic polarity involves three distinct phases of kinocilium movement (Tarchini et al., 2013): (1) initial off-centre migration toward the lateral edge of the hair cell, (2) positioning within a crescent defined by Gpsm2/Gαi activity, and (3) final relocation—together with the maturing stereociliary bundle—toward the cell centroid as the bare zone forms.

Our data indicate that the intrinsic polarity defect in Pcdh15-CD2 mutants arises specifically during step 2: the kinocilium fails to localise at the centre of the Gpsm2/Gαi crescent. This process occurs between E16.5 and E17.5 (Tarchini et al., 2013). It is unlikely that this positioning is initiated through kinocilium–stereocilium adhesion, as these structures remain physically distant at this stage. Furthermore, in our rescue experiment, it is difficult to envision how the kinocilium would recouple with the stereocilia through adhesion alone (that too over the large gap developed in Pcdh15-n38 mutant HC – a link is around 50 nm and the gap that forms in mutants between the kinocilia and the tallest stereocilia can range from 1-4 µm) if that were the exclusive mechanism involved. However, to confirm the timing of kinociliary link formation, we contacted Dr. Peter Barr-Gillespie, who confirmed via personal communication that kinocilial links are not present at E16.5. We observe clear kinocilium positioning defects in Pcdh15-CD2 mutants as early as E16.5, prior to the onset of kinociliary link formation. These early defects, together with the similarity of the Pcdh15-CD2 phenotype to that of ciliary gene mutants, strongly support a distinct and earlier role for PCDH15-CD2 in intrinsic polarity regulation, independent of its later function in kinocilial adhesion.

That said, we agree that kinocilial links likely become functionally important during the final repositioning phase. As the Gpsm2/Gαi domain expands and the stereociliary bundle shifts toward the centroid of the cell, kinocilial links likely anchor the kinocilium to the tallest stereocilium. In Pcdh15n38/n38 mutants, the absence of PCDH15-CD2 prevents this anchoring, resulting in the kinocilium being left behind during bundle repositioning.

We have revised the Discussion to clarify the developmental timing of these processes and to propose a biphasic model in which PCDH15-CD2 functions first in intrinsic polarity establishment, and later in kinocilial anchoring during final bundle repositioning.

*To their credit, they now include additional measures of hair cell orientation based on stereociliary alignment and compare them with kinocilium-based measures. They demonstrate that these two methods reveal different phenotype severities—an important observation that underscores how divergent interpretations can result from the selection of different phenotyping criteria. This discrepancy warrants explicit discussion, particularly regarding how "disruption" is defined and by what criteria. It also reinforces my own interpretation that the primary function of Pcd15-CD2 is kinocilium:stereocilia linkage and that any impacts on intrinsic hair cell polarity are indirect.*

We thank the reviewer for these suggestions during the first round of revision.

We propose that the key divergence arises from a disruption in step 2 of intrinsic polarity establishment, when the kinocilium positions itself in register with the Gαi domain. As others have shown, the Gαi domain defines the site of stereocilia generation. The pathways that specify the kinocilium and the Gai domain are different, and if not coupled, one would expect the variations in the alignment of the stereocilia and the kinocilia to be different. We believe that the failure to couple the kinocilium to the stereocilia in the mutants underlies the difference between the two phenotypes. Our data support a model where the failure of the kinocilium to move into the Gai crescent results from an early requirement for Pcdh15-CD2 in the kinocilium/basal body.

It is important to note that these measurements were conducted at P0, as earlier-stage stereocilia bundles lack sufficient definition to reliably determine the apex. By P0, the hair bundle has repositioned toward the cell centroid. In the mutant context, failure to properly anchor the kinocilium to the stereociliary bundle likely exacerbates the divergence in positioning at this stage.

We have revised the Discussion to incorporate these points and clarify our interpretation.

*Regarding my second point, it's notable—if not puzzling—that the adhesive role of Pcd15-CD2 receives little to no attention in the discussion. Given the protein’s known function in kinocilium–stereocilium cohesion, the authors should clearly articulate why they are interpreting it to have a direct role in intrinsic polarity regulation instead, and why readers like myself should reconsider their existing expectations. A few well-placed sentences in the Discussion section would assuage my contrary interpretation.*

We thank the reviewer for this suggestion. As recommended, we have revised the Discussion to include the role of PCDH15-CD2 in the formation of kinocilial links and its contribution to the refinement of apical hair cell architecture—specifically during the final repositioning of the hair bundle toward the centre of the cell.

*Reviewer #3: The authors did a good job addressing my questions.*

We thank the reviewer for their suggestions during the first round of revision and appreciate the above comment.

*One minor point: "polarity" in the Keywords section was misspelt*.

We thank the reviewer for pointing this out. We have corrected it in the revised manuscript.
